# Inflammatory Bowel Disease Self-Care Behaviors in Context of Psychosocial Factors

**DOI:** 10.1007/s10620-025-09326-y

**Published:** 2025-08-12

**Authors:** Jordan Anders-Rumsey, Lauren Kasmikha, Melody Chiang, Gal Hodish, Jessica Sheehan, Jessica R. Golbus, Ulrica Loven Wickman, Andrew Admon, Kenneth Resnicow, John Sturgeon, Shirley Cohen-Mekelburg

**Affiliations:** 1 Department of Internal Medicine, University of Michigan Medicine, Ann Arbor, MI USA; 2 University of Michigan Medical School, Ann Arbor, MI USA; 3 Division of Gastroenterology & Hepatology, University of Michigan Medicine, 3912 Taubman Center, 1500 E. Medical Center Drive, Ann Arbor, MI 48109 USA; 4 Division of Cardiology, University of Michigan Medicine, Ann Arbor, MI USA; 5 Department of Health and Caring Sciences, Linnaeus University, Växjö, Sweden; 6 Division of Pulmonary and Critical Care Medicine, University of Michigan Medicine, Ann Arbor, MI USA; 7 VA Center of Clinical Management Research, VA Ann Arbor Healthcare System, Ann Arbor, USA; 8 School of Public Health, University of Michigan, Ann Arbor, MI USA; 9 Department of Anesthesiology, University of Michigan Medicine, Ann Arbor, MI USA

**Keywords:** Crohn’s disease, Ulcerative colitis, Self-management, Confidence

## Abstract

**Background:**

IBD self-management behaviors can be classified into three components: monitoring, adaptation, and maintenance.

**Aims:**

Our primary objective was to understand variation in IBD self-management behaviors. Our secondary objective was to examine the relationship between disease burden, self-efficacy, and these IBD self-management behaviors.

**Methods:**

We conducted a prospective survey study of patients with IBD using the IBD self-care questionnaire, treatment self-regulation questionnaire, IBD self-efficacy scale, understanding IBD questionnaires, and ulcerative colitis (UC)/Crohn’s disease patient-reported outcome measures. We examined the relationship between IBD monitoring, adaptation, maintenance behaviors, and both IBD self-efficacy and current disease burden using multivariable ordinal logistic regression.

**Results:**

We enrolled 87 participants (mean age, 55.2 [sd = 17.1] years, 51.7% female, 47.1% with UC) who completed survey questions. Overall participation in IBD monitoring, adaptation, and maintenance behaviors was common. In a multivariable analysis, higher self-efficacy was associated with more frequent monitoring of non-inflammatory symptoms after adjusting for disease burden, age, sex and IBD type (odds ratio[OR] = 1.013, *p* = 0.006). Separately, higher disease burden was associated with more frequent adaptation (including avoiding sex, OR = 1.089, *p* < 0.001; avoiding activities, OR = 1.079, *p* < 0.001; planning around bathrooms, OR = 1.090, *p* < 0.001; planning around IBD, OR = 1.127, *p* < 0.001).

**Conclusion:**

Study findings highlight a complex relationship between IBD self-management behaviors, self-efficacy, and disease burden. Self-management support strategies that adapt to the needs of patients at times of high disease burden may offer unique benefits. Further work is needed to explore how best to adapt these strategies into effective interventions.

**Supplementary Information:**

The online version contains supplementary material available at 10.1007/s10620-025-09326-y.

## Introduction

Inflammatory bowel disease (IBD) is a group of disorders characterized by chronic inflammation of the gastrointestinal tract, with ulcerative colitis and Crohn’s disease being the most common. These conditions affect over 6.8 million people worldwide, leading to substantial disability [1, 2]. While gastroenterologists play a principal role in treating patients with IBD, preventing complications, and educating patients, most IBD management occurs between clinic visits, shifting the responsibility of day-to-day management to patients [3–5]. Indeed, higher engagement in IBD self-management is associated with fewer symptoms and improved quality of life [6–9]. Self-management behaviors can be classified into three components: monitoring, adaptation, and maintenance [10]. Monitoring involves recognizing and observing changes in one’s health (i.e., symptom changes). Adaptation involves adjusting behavior to improve symptoms (i.e., avoiding triggering foods or strenuous activity). Lastly, maintenance aims to sustain health and prevent exacerbation (i.e., medication adherence, stress management) [11].

**Fig. 1:**
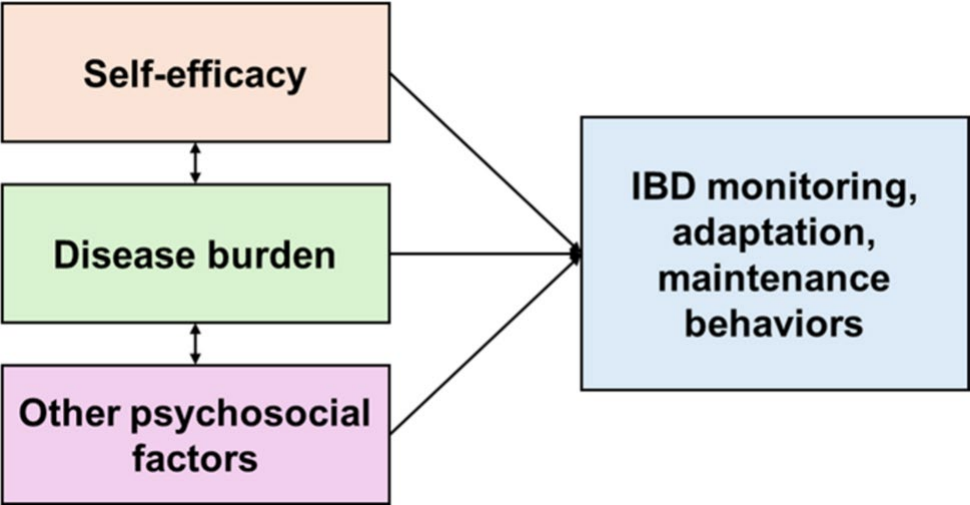
Conceptual model of the relationship between self-efficacy, disease, burden, other psychosocial factors and IBD self-care behaviors

The role of self-management is well supported for a range of chronic conditions and has been associated with improved health outcomes. For example, among patients with heart failure, more frequent engagement with these three types of behaviors led to improved health-related quality of life [12–14], fewer hospitalizations [15–18], and lower mortality [19, 20]. Self-management is similarly likely to be a key determinant of health outcomes for patients with IBD. In a Swedish IBD cohort, monitoring, adaptation, and maintenance behaviors were more common among patients with more active IBD. [21] However, it is unclear which key factors contribute to patient engagement in self-management. Lastly, self-efficacy, which is an individual’s confidence in their ability to perform a specific behavior or skill, has historically been linked to greater engagement in that self-management behavior, according to Bandura’s Social Cognitive Theory (Fig. 1). [22] Our prior work found that higher IBD-related self-efficacy was associated with a lower IBD symptom burden, consistent with established theory [23]. However, the relationship between self-efficacy and monitoring, adaptation, and maintenance behaviors is not well known. Therefore, our primary objective was to understand variation in IBD self-management behaviors. Our secondary objective was to examine the relationship between disease burden, self-efficacy, and these IBD self-management behaviors.

## Methods

### Study Design

We conducted a prospective observational study of patients with IBD (based on three or more diagnostic codes) receiving care at a tertiary IBD referral center. Patients were eligible if they had IBD and an available email or phone number to facilitate enrollment. We excluded patients under 18 years, those without the capacity to consent, and non-English speakers. Study recruitment was facilitated by e-mail invitation and two reminder emails at two-week intervals followed by telephone calls to patients who did not opt out of participation. All study procedures were approved by the Institutional Review Board (HUM#00255816).

### Measures

Participants were asked to complete the IBD Self-Care Questionnaire (SCQ) [21], Treatment Self-Regulation Questionnaire (TSRQ) [24–29], IBD Self-Efficacy Scale (IBD-SES) [30], Understanding IBD Questionnaires (UIBDQ) [31], and ulcerative colitis (UC) or Crohn’s disease (CD) Patient-Reported Outcome measures (UC-PRO or CD-PRO) [32, 33] (Supplemental Fig. 1). The 22-item IBD-SCQ measures the frequency at which patients complete IBD monitoring, adaptation, and maintenance behaviors and is scored on a “never” to “always” scale. The shortened 8-item TSRQ measures autonomous and controlled motivation for medication adherence, managing stress and emotions, communicating with clinicians, and symptom monitoring and is scored on a 1 to 5 scale, with higher scores indicating greater endorsement of each motivation type (e.g., autonomous, controlled). The 29-item IBD-SES assesses confidence in managing stress and emotions, medical care, symptoms and disease, and remission. The IBD-SES is scored using a 1 to 10 scale, with 1 indicating “not at all confident” and 10 indicating “totally confident.” Total scores range from 29 to 290, with higher scores indicating greater self-efficacy. The 36-item UIBDQ measures patients’ IBD-specific knowledge across eight topics. The UIBDQ is scored on a 0 to 36 scale, with a higher mean score indicating higher IBD knowledge. Finally, the 9-item UC-PRO and CD-PRO daily impact scale was used as a surrogate for disease burden and is scored on a 1 to 5 scale, with higher scores indicating greater IBD daily life impact.

### Analytic Plan

Spearman correlations with a *p* < 0.10 between monitoring, adaptation, maintenance behaviors and either IBD self-efficacy or current disease burden were included in multivariable ordinal logistic regression models. For the models, the dependent variable was the specific behavior, and both self-efficacy and disease burden were key independent variables, with age, sex, and IBD type as covariates. Statistical analysis was performed in Stata (StataCorp, Inc. College Station, TX).

## Results

### Cohort Identification and Demographics

We identified 728 patients with three or more IBD diagnostic codes. Among them, 20 patients had erroneous emails, 49 reported no diagnosis of IBD, and 25 were deceased, resulting in 634 patients meeting inclusion criteria. Of these, 14% of the 634 patients completed the survey. Therefore, 87 participants comprised the study cohort. Participants had a mean age of 55.2 (sd = 17.1) years, 45 (51.7%) were female, and 85 (97.7%) were White. With respect to IBD type, 42 (48.3%) had CD, 41 (47.1%) had UC, and 4 (4.6%) had IBD unclassified (Table 1). Participant motivation and self-efficacy varied (Table 2).

**Table 1:** Demographic and disease-specific characteristics of the study cohort

Demographics	*n *= 87
Age (mean, sd)	55.2 (17.15)
Sex (*n*, %)	
Male	42 (48.3%)
Female	45 (51.7%)
Race (*n*, %)	
White	84 (96.6%)
Black	2 (2.3%)
Other	1 (1.1%)
Ethnicity (*n*, %)	
Non-Hispanic	76 (87.4%)
Hispanic	3 (3.4%)
Other or unknown	8 (9.2%)
Location of residence (*n*, %)	
Urban	12 (13.5%)
Suburban	51 (57.3%)
Rural	26 (29.2%)
Education (*n*, %)	
Less than high school	1 (1.2%)
High school	8 (9.2%)
Some college	15 (17.2%)
Associate degree	13 (14.9%)
Bachelor degree	25 (28.7%)
Graduate degree	25 (28.7%)
IBD Characteristics	
IBD Type (*n*, %)	
Ulcerative Colitis	41 (47.1%)
Crohn’s disease	42 (48.3%)
IBD unclassified or unknown	4 (4.6%)
Crohn’s disease phenotypes (*n*, %)	
Stricturing	5 (11.9%)
Fistulizing	9 (21.4%)
Non-stricturing non-fistulizing	13 (31.0%)
Unknown phenotype	15 (35.7%)
Crohn’s disease extent (*n*, %)	
Colonic involvement only	8 (19.1%)
Small bowel involvement only	7 (16.7%)
Small bowel and colon involvement	26 (61.9%)
Upper gastrointestinal involvement	7 (16.7%)
Ulcerative colitis or IBD-U extent (*n*, %)	
Proctitis	6 (13.3%)
Left-sided colitis	13 (28.9%)
Extensive colitis	21 (46.7%)
Unknown extent	5 (11.1%)
Medications (*n*, %)	
Oral steroids	7 (8.1%)
5-ASA	18 (20.7%)
MTX	3 (3.5%)
Azathioprine	6 (6.9%)
Anti-tumor necrosis factor alpha	23 (26.4%)
Anti integrins	9 (10.3%)
Anti-interleukin 12/23	9 (10.3%)
Janus Kinase inhibitors	5 (5.8%)
No current IBD-targeted therapy	23 (26.4%)
Comorbidities (*n*, %)	
Anxiety	36 (41.9%)
Depression	31 (36.9%)

**Table 2:** Summary statistics for participant motivation, self-efficacy, and IBD knowledge

Motivation for…	Mean scores (sd)
Taking IBD-specific medications	
Total motivation	3.91 (1.46)
Autonomous motivation	4.66 (1.31)
Controlled motivation	3.42 (1.78)
Managing stress and mood	
Total motivation	3.88 (1.51)
Autonomous motivation	4.77 (1.59)
Controlled motivation	3.32 (1.76)
Communicating with the medical team about IBD-related symptoms or issues	
Total motivation	3.87 (1.81)
Autonomous motivation	4.67 (1.70)
Controlled motivation	3.29 (2.03)
Paying attention to and monitoring gastrointestinal symptoms regularly	
Total motivation	3.79 (1.68)
Autonomous motivation	4.77 (1.49)
Controlled motivation	3.09 (1.93)

Self-efficacy in…	Mean (sd)
Managing stress and emotions	54.68 (19.38)
Managing medications	68.41 (15.82)
Managing symptoms and disease	44.08 (14.73)
Managing remission	34.99 (9.72)
Total IBD self-efficacy	199.95 (50.42)

IBD-specific knowledge	Mean (sd)
Crohn’s disease totals	90.8 (41.7)
Physiology	10.4 (4.5)
Diagnostics	6.3 (3.3)
Medications	8.7 (5.5)
Surgery	8.6 (5.2)
Preventative Care	6.1 (3.4)
Nutrition	5.6 (2.0)
Diet	5.0 (3.7)

Current IBD daily impact	
Overall	5.28 (7.03)
Crohn’s disease specific	4.01 (7.24)
Ulcerative colitis specific	6.55 (7.79)

### Self-Management Behaviors

Overall participation in IBD monitoring, adaptation, and maintenance behaviors was common (Table 3). Participants reported more frequently monitoring intestinal symptoms as compared to non-intestinal or psychological symptoms. A smaller but substantial subset of the sample reported adapting their diet. Activity restrictions were less common, with alcohol being the most commonly avoided. At times, participants planned their day near a toilet or in view of their disease. Patients reported monitoring sleep and stress, but their engagement in specific treatment modalities was much more variable. Participants generally endorsed taking their medications as prescribed and knew whom to contact with symptom changes. Most patients reported that self-management behaviors relieved their IBD symptoms.

**Table 3:** Summary of participant self-management behaviors

	Never	Sometimes	Often	Always	Not Applicable
Monitoring behavior					
I pay attention to intestinal symptoms	4 (4.6%)	2 (2.3%)	17 (19.5%)	64 (73.6%)	
Physical symptoms unrelated to the intestines	7 (8.1%)	7 (8.1%)	20 (23.3%)	49 (57.0%)	3 (3.5%)
Psychological symptoms	13 (15.3%)	14 (16.5%)	18 (21.2%)	37 (43.5%)	3 (3.5%)
How often do you feel confident that you are able to determine whether intestinal symptoms are due to inflammatory bowel disease?	5 (5.8%)	14 (16.1%)	43 (49.4%)	25 (28.7%)	
Adaptation behavior					
I adapt my diet	9 (10.3%)	10 (11.5%)	36 (41.4%)	31 (35.6%)	1 (1.2%)
I avoid various activities	25 (28.7%)	24 (27.6%)	24 (27.6%)	14 (16.1%)	
I avoid sex	38 (44.2%)	18 (20.9%)	13 (15.1%)	7 (8.1%)	10 (11.6%)
I avoid alcohol	19 (22.1%)	14 (16.3%)	16 (18.6%)	30 (34.9%)	7 (8.1%)
I plan my day so that I am always near a toilet	28 (32.2%)	19 (21.8%)	22 (25.3%)	18 (20.7%)	
I plan my day in view of the fact that I have inflammatory bowel disease	29 (33.3%)	18 (20.7%)	19 (21.8%)	21 (24.1%)	
Maintenance behavior					
Self-care to make sure I sleep well	6 (6.9%)	12 (13.8%)	37 (42.5%)	31 (35.6%)	1 (1.2%)
Self-care to manage stress	7 (8.1%)	15 (17.2%)	43 (49.4%)	22 (25.3%)	
I find out more about inflammatory bowel disease	14 (16.1%)	16 (18.4%)	31 (35.6%)	25 (28.7%)	1(1.2%)
I look for new approaches to living with inflammatory bowel disease	21 (24.1%)	19 (21.8%)	23 (26.4%)	23 (26.4%)	1(1.2%)
I use natural remedies	32 (36.8%)	24 (27.6%)	19 (21.8%)	10 (11.5%)	2 (2.3%)
I perform other self-care	30 (34.5%)	4 (4.6%)	31 (35.6%)	8 (9.2%)	14 (16.1%)
		No	Yes		
Do you take medication for inflammatory bowel disease as prescribed by a doctor?		20 (23.0%)	67 (77.0%)		
Skills		No	Yes		
Do you know whom to contact if you experience symptoms of inflammatory bowel disease?		10 (11.5%)	77 (88.5%)		
	Not familiar	–	–	Familiar	
How familiar are you with the symptoms for which you should contact a healthcare provider?	3 (3.5%)	10 (11.5%)	28 (32.2%)	46 (52.9%)	
	Daily	–	–	Never	
Do you smoke?	5 (5.8%)	1 (1.2%)	3 (3.5%)	78 (89.7%)	
Do you use snuff?		1 (1.2%)		86 (98.9%)	
How often does self-care help you relieve symptoms of inflammatory bowel disease?	9 (10.3%)	23 (26.4%)	36 (41.4%)	7 (8.1%)	12 (13.8%)

### Self-Efficacy and Disease Burden

On examination of Spearman correlations, self-efficacy positively correlated with avoiding various activities (rho = 0.50, *p* = 0.0001), avoiding sex (rho = 0.48, *p* = 0.0005), planning around bathrooms (rho = 0.48, *p* = 0.0002), and planning around IBD (rho = 0.54, *p* < 0.0001). On multivariable ordinal logistic regressions, higher self-efficacy was significantly associated with more frequent monitoring of non-inflammatory symptoms after adjusting for disease burden, age, sex, and IBD type (odds ratio[OR] = 1.013, *p* = 0.006). In separate multivariable ordinal logistic regressions, higher disease burden was significantly associated with more frequent adaptation (including avoiding sex, OR = 1.089, *p* < 0.001; avoiding activities, OR = 1.079, *p* < 0.001; planning around bathrooms, OR = 1.090, *p* < 0.001; planning around IBD, OR = 1.127, *p* < 0.001) after adjusting for self-efficacy, age, sex, and IBD type (Table 4).

**Table 4:** Associations between participant self-care behaviors, self-efficacy, and disease burden

Dependent variable	Independent variable	Odds ratio	95% Confidence intervals	*p* value
Monitoring: unrelated physical symptoms	Self-efficacy	1.013	1.003, 1.022	0.006
	Disease burden	1.024	0.986, 1.064	0.216
Adaptation: sex	Self-efficacy	0.991	0.982, 1.001	0.067
	Disease burden	1.089	1.043, 1.137	< 0.001
Adaptation: diet	Self-efficacy	0.992	0.983, 1.001	0.068
	Disease burden	1.037	0.999, 1.076	0.056
Adaptation: activities	Self-efficacy	0.996	0.987, 1.005	0.339
	Disease burden	1.079	1.038, 1.123	< 0.001
Adaptation: planning around bathroom	Self-efficacy	0.995	1.046, 1.137	0.225
	Disease burden	1.090	1.046, 1.137	< 0.001
Adaptation: planning around IBD	Self-efficacy	0.995	0.986, 1.004	0.249
	Disease burden	1.127	1.073, 1.184	< 0.001

## Discussion

Our findings revealed the most commonly endorsed self-management behavior domains involved symptom monitoring, whereas patient engagement in maintenance and adaptation behaviors was more heterogeneous. This pattern suggests a high degree of commitment among respondents to tracking symptoms and less consistent downstream behavioral responses (e.g., adapting diet). Importantly, a majority of the sample endorsed regular monitoring not only of intestinal symptoms but also non-intestinal and psychological symptoms. Further, they endorsed benefit from self-management of other factors like stress and sleep disturbance. Interestingly, respondents endorsed more frequent self-care behaviors than in a prior Swedish IBD study. The most notable differences centered on monitoring behaviors: 28% of the Swedish cohort reported always paying attention to intestinal symptoms as compared to 74% of our cohort, and only 13% of the Swedish cohort reported always paying attention to non-intestinal symptoms as compared to 57% in our cohort [34]. Nonetheless, both cohorts reported similar levels of confidence distinguishing IBD from non-IBD symptoms and maintained the perception that self-care helped their symptoms. Therefore, these differences may potentially relate to cultural factors or differences in study period.

Participants endorsing higher self-efficacy reported more frequent non-inflammatory symptom monitoring. However, self-efficacy was not meaningfully related to adaptation or maintenance behavior. In contrast, higher IBD burden was associated with more frequent adaptation. Monitoring symptoms is a key driver of self-efficacy according to Bandura’s theory of self-efficacy [35]. Conversely, higher self-efficacy empowers patients to monitor their symptoms and can lower symptom burden [23]. Self-efficacy can be effectively targeted through cognitive behavioral theory interventions [36]. These skills can be useful in both active and inactive disease states to help patients in action planning and managing symptoms. Identification of self-efficacy as a key psychosocial factor builds on previously published literature to further support promotion of self-efficacy as a target for IBD self-management programs, particularly to promoting symptom monitoring [23]. However, promoting self-efficacy may not be sufficient to affect adaptation and maintenance behaviors.

Patients with IBD often describe a need for tailoring with less care needed during times of remission and more care during times of flare. Symptoms also drive patients to seek alternative treatments [37]. Our study findings that higher IBD burden was associated with more frequent self-care adaptation support this trend, as did Swedish cohort findings [34]. Perceived severity of disease can influence health behaviors according to the health belief model [38]. Our findings may reflect adaptation of behavior in response to higher disease burden in an attempt to alleviate symptoms. This suggests potential for interventions that can be tailored to the needs of patients with IBD when they need them most—in times of higher disease burden. Further, resolution of avoidance behavior (i.e., participating in desired activities) may be a goal of patient-centered care to improve quality of life.

This study’s strengths include use of validated measures and an IBD cohort with variation in disease characteristics, motivation, and self-efficacy. However, the single-center nature of this study may limit study generalizability, and response rate may lead to selection bias. Further, it would be interesting to correlate clinician-rated disease burden with patient-reported disease impact in future work. In addition, as eHealth tools grow in popularity, it will also be interesting to better understand their influence on self-efficacy and self-management behaviors in future work. Finally, self-management behaviors are measured by self-report rather than observed, and confounders to self-management may persist in the context of relatively low power.

In conclusion, our results highlight a complex relationship between self-management behaviors, self-efficacy, and IBD symptom burden. Individuals with high IBD-related self-efficacy endorsed high overall levels of symptom monitoring, but not meaningful increases in adaptation or maintenance behaviors. These latter domains, in contrast, were more meaningfully associated with IBD-related burden. Self-management support strategies that provide direct feedback on performance and adapt to the needs of patients at times of higher disease burden may offer unique benefits, including through psychological support, patient education, and skill building. Qualitative work to explore how best to adapt these strategies to patients’ needs would help to translate these findings into effective interventions.

## Supplementary Information

Below is the link to the electronic supplementary material.

Supplementary file1 (DOCX 23 KB)

## Data Availability

The data that support the findings of this study are available on reasonable request by emailing the corresponding author.
