# Supplementary material for: Inflammatory Bowel Disease Self-Care Behaviors in Context of Psychosocial Factors
Source: Dig Dis Sci. Author manuscript; Available in PMC 2025 Sep 9. (PMC12416496; doi:10.1007/s10620-025-09326-y)
Supplement: Supplementary file 1 [file NIHMS2106492-supplement-Supplemental_Figure_1.docx]

**Supplemental Table 1: Consolidated Survey Items**

| **IBD Details** |
| --- |
| 1. Do you carry a diagnosis of inflammatory bowel disease such as Crohn's disease or ulcerative colitis? Yes No 2. What is your Inflammatory bowel disease (IBD) type  Ulcerative colitis  Crohn’s disease  Indeterminate colitis  Unknown 3. What medications have you taken in the past? [all that apply]  Steroids (prednisone or budesonide)  Mesalamine  Methotrexate  Azathioprine/6-mercaptopurine  Anti-tumor necrosis factor agents (infliximab, adalimumab, certolizumab, golimumab)  Anti-integrin (vedolizumab)  Anti-IL12/23 (ustekinumab)  Tofacitinib 4. What medications are you currently taking? [all that apply]  Steroids (prednisone or budesonide)  Mesalamine  Methotrexate  Azathioprine/6-mercaptopurine  Anti-tumor necrosis factor agents (infliximab, adalimumab, certolizumab, golimumab)  Anti-integrin (vedolizumab)  Anti-IL12/23 (ustekinumab)  Tofacitinib |
| **IBD Self-Management** |
| 1. **Do you pay attention to symptoms of inflammatory bowel disease?** *Scored: from 1 (never) to 4 (always) and 5 (not applicable)* I pay attention to intestinal symptoms [     ]   Physical symptoms unrelated to the intestines [     ]   Psychological symptoms [     ]   2. **How often do you feel confident that you are able to determine whether intestinal symptoms are due to inflammatory bowel disease?** *Scored: from 1 (never) to 4 (always) and 5 (not applicable)* [     ]   3. **How often do you adapt your day to problems caused by inflammatory bowel disease?** *Scored: from 1 (never) to 4 (always) and 5 (not applicable)* I adapt my diet  [     ]    I avoid various activities [     ]   I avoid sex [     ]   I avoid alcohol   [     ]    I plan my day so that I am always near a toilet  [     ]   I plan my day in view of the fact that I have inflammatory bowel disease  [     ]   4. **How often do you administer self-care to prevent or relieve symptoms of inflammatory bowel disease?** *Scored: from 1 (never) to 4 (always) and 5 (not applicable)* Self-care to make sure I sleep well [     ]    Self-care to manage stress  [     ]   I find out more about inflammatory bowel disease  [     ]   I look for new approaches to living with inflammatory bowel disease  [     ]   I use natural remedies  [     ]    I perform other self-care  [     ]    5. **Do you take medication for inflammatory bowel disease as prescribed by a doctor?** *Scored: from 1 (no) and 2 (yes)* [     ]   6. **Do you know whom to contact if you experience symptoms of inflammatory bowel disease?** *Scored: from 1 (no) and 2 (yes)* [     ]   7. **How familiar are you with the symptoms for which you should contact a healthcare provider?** *Scored from: 1 (not familiar) to 4 (totally familiar)* [     ]   8. **Tobacco use:** *Scored from: 1 (daily use) to 4 (never)* Do you smoke?  [     ]   Do you use snuff?  [     ]   9. **How often does self-care help you relieve symptoms of inflammatory bowel disease?** *Scored: from 1 (never) to 4 (always) and 5 (not applicable)*  [     ] |
| **Motivation in IBD** |
| There are a variety of reasons why patients take their medications, monitor their symptoms, manage their mood, and communicate with their health care team. Please consider the following behaviors and on a 1-7 scale, if 1 is not at all true and 7 is very true, indicate how true each of these reasons is for you. **1. I take my IBD-specific medications because:** (If you do not take IBD-specific mediations please skip to question 2) a. Other people would be mad at me if I didn’t. [     ]   b. I find it a personal challenge to do so. [     ]   c. I personally believe that controlling my IBD will improve my health. [     ]   d. I would feel guilty if I didn’t do what my doctor said. [     ]   e. I want my doctor to think I’m a good patient. [     ]   f. I would feel bad about myself if I didn’t. [     ]   g. It’s exciting to try to keep my IBD in a healthy range. [     ]   h. I don’t want other people to be disappointed in me. [     ]   **2. I manage my stress and mood because:** (If you do not manage your stress and mood, please skip to question 3) a. Other people would be mad at me if I didn’t. [     ]   b. I find it a personal challenge to do so. [     ]   c. I personally believe that controlling my IBD will improve my health. [     ]   d. I would feel guilty if I didn’t do what my doctor said. [     ]   e. I want my doctor to think I’m a good patient. [     ]   f. I would feel bad about myself if I didn’t. [     ]   g. It’s exciting to try to keep my IBD in a healthy range. [     ]   h. I don’t want other people to be disappointed in me. [     ]   **3. I communicate with my medical team when I have IBD-related symptoms or issues because:** (If you do not communicate with your medical team, please skip to question 4) a. Other people would be mad at me if I didn’t. [     ]   b. I find it a personal challenge to do so. [     ]   c. I personally believe that controlling my IBD will improve my health. [     ]   d. I would feel guilty if I didn’t do what my doctor said. [     ]   e. I want my doctor to think I’m a good patient. [     ]   f. I would feel bad about myself if I didn’t. [     ]   g. It’s exciting to try to keep my IBD in a healthy range. [     ]   h. I don’t want other people to be disappointed in me. [     ]   **4. I pay attention to and monitor my gastrointestinal symptoms regularly because:** (If you do not pay attention to and monitor your gastrointestinal symptoms please move to next survey) a. Other people would be mad at me if I didn’t. [     ]   b. I find it a personal challenge to do so. [     ]   c. I personally believe that controlling my IBD will improve my health. [     ]   d. I would feel guilty if I didn’t do what my doctor said. [     ]   e. I want my doctor to think I’m a good patient. [     ]   f. I would feel bad about myself if I didn’t. [     ]   g. It’s exciting to try to keep my IBD in a healthy range. [     ]   h. I don’t want other people to be disappointed in me. [     ] |
| **Confidence and IBD** |
| **29-item IBD Self-Efficacy Scale (**Keefer et al.**)** **Over the past 2 weeks, how confident have you felt in your ability to perform each of the following tasks?** **1** **2** **3** **4** **5** **6** **7** **8** **9** **10** **not confident at all** **some what confident** **totally confident** **Managing your stress and emotions**  1. Keep from getting stressed?  2. Do something to make yourself less stressed?  3. Keep from getting discouraged?  4. Do something to make yourself feel better when discouraged?  5. Keep from feeling sad or down in the dumps?  6. Do something to make yourself feel better when sad?  7. Keep sadness or anxiety from interfering?  8. Do something to make yourself feel better when your sadness or anxiety interferes?  9. Get emotional support from family or friends? **Managing your medical care**  10. Follow the instructions for your prescription medications?  11. Take your prescription medication at the appropriate times?  12. Take the medications to prevent a flare up of your IBD as directed?  13. Work with your doctor or nurse to reach an agreement on a treatment plan?  14. Ask your doctor about your illness?  15. Discuss openly with your doctor any problems related to your medications?  16. Work out differences with your doctor?  17. Ask your doctor about your medications? **Managing your symptoms and disease**  18. Reduce your symptoms in general?  19. Keep sleep problems from interfering?  20. Keep physical discomfort or pain from interfering?  21. Keep diarrhea and/or urgency from interfering?  22. Keep any other symptoms or health problems you have from interfering?  23. Decrease your fatigue?  24. Keep fatigue from interfering? **Maintaining remission**  25. Manage your disease in general?  26. Keep your disease in remission?  27. Engage in self-care? (exercise, rest, diet, etc.)  28. Engage in/continue with a stress management program?  29. Maintain your sense of well-being? |
| **Demographics** |
| 1. What is your age? 2. What is your sex? 3. What is your race? [all that apply] 4. What is your ethnicity? 5. What is the highest level of school you have completed or the highest degree you have received? 6. In addition to inflammatory bowel disease, do you carry a diagnosis of: [yes/no]  Depression  Anxiety  Arthritis (rheumatoid or psoriatic)  Ankylosing spondylitis  Hydradenitis suppurativa 7. Where is your home located?  Urban  Suburban  Rural |
